# PFAS Exposure Induces Hearing Loss by Targeting Cochlear Hair Cells, Ribbon Synapses, and Spiral Ganglions

**DOI:** 10.21203/rs.3.rs-7244874/v1

**Published:** 2025-09-02

**Authors:** Pankaj Bhatia, Katherine Roth, Zhao Yang, Michael Petriello, Samson Jamesdaniel

**Affiliations:** Wayne State University; Wayne State University; Wayne State University; Wayne State University; Wayne State University

**Keywords:** PFAS-induced hearing loss, cochlear synaptopathy, outer hair cell loss, spiral ganglions

## Abstract

Background Per- and polyfluoroalkyl substances (PFAS) are persistent environmental pollutants linked to adverse health effects. Recent epidemiological data suggest an association between PFAS exposure and hearing impairment, but underlying mechanisms remain unclear. This study examined PFAS-induced auditory dysfunction using Ldlr^−^/^−^ mice on a C57BL/6J background exposed for seven weeks to a mixture of five PFAS compounds (2 mg/L each) in drinking water. PFAS exposure significantly elevated hearing thresholds by 18–33 dB across multiple frequencies, indicative of auditory impairment. Functional assessments revealed impaired outer hair cell (OHC) function, and immunohistochemical analysis identified ~ 24% OHC loss in the basal turn of the cochlea. In addition, PFAS exposure reduced wave-I amplitudes and increased latencies, suggesting cochlear synaptopathy. Immunohistochemistry further demonstrated a significant decrease in ribbon synapse numbers (CtBP2 and GluR2 markers) per inner hair cell and a ~ 53% reduction in spiral ganglion cell density. Overall, PFAS exposure induced cochlear synaptopathy and high-frequency hearing loss in mice. The findings also indicated that cochlear OHCs, ribbon synapses, and spiral ganglions are potential targets in PFAS-induced hearing loss. Together, these data suggest that PFAS exposure elicits a multifaceted ototoxic response, affecting both sensory and neural elements of the cochlea.

## Introduction

Environmental exposure to per- and polyfluoroalkyl substances (PFAS) poses serious risks to human health, particularly for neonates, children, and older adults [1–4]. PFAS comprise a vast category of man-made chemicals, totaling thousands, that have been extensively utilized across multiple industries since the 1950s. PFAS are widely used in nonstick cookware, waterproof clothing, food packaging materials, and fire-fighting foams [5–9]. They do not break down easily and pollute the environment and the human body, resulting in widespread exposure [10–12]. PFAS are found in almost 4% of water supplies in the United States, impacting over 110 million people. Additionally, about 98% of American adults have detectable blood levels of PFAS, linked to various health impacts [2, 13].

Exposure to PFAS is linked to a broad range of adverse health effects, including increased risks of various cancers, such as kidney, testicular, and prostate cancers and cardiometabolic diseases. It can also lead to diminished immune response; developmental delays in children, like low birth weight, early puberty, and behavioral abnormalities; Hormonal and metabolic disturbances, such as elevated cholesterol, liver disease, and disruption of thyroid function and lipid metabolism; problems with reproduction; and neurotoxicity, specifically impacting dopamine and glutamate-based neurotransmission systems [3, 4, 14–31]. The Nervous system is highly susceptible to PFAS toxicity. Recent research shows a link between PFAS and neurodegenerative diseases, including Parkinson’s disease and Alzheimer’s disease. PFAS have been found to cross the blood-brain barrier and accumulate in brain tissue, potentially contributing to the development and progression of these disorders [32, 33]. PFAS exposure has been shown to disrupt synaptic neurotransmission [33], potentially through mechanisms involving dopamine-glutamate imbalance and calcium dysregulation [34–38]. Additionally, PFAS induces oxidative stress via excessive reactive oxygen species (ROS) generation [39] and depletion of antioxidant defenses, while also promoting neuroinflammation through microglial activation and disruption of the blood-brain barrier [40], which increases the risk of neurodegeneration.

**Figure 1:**
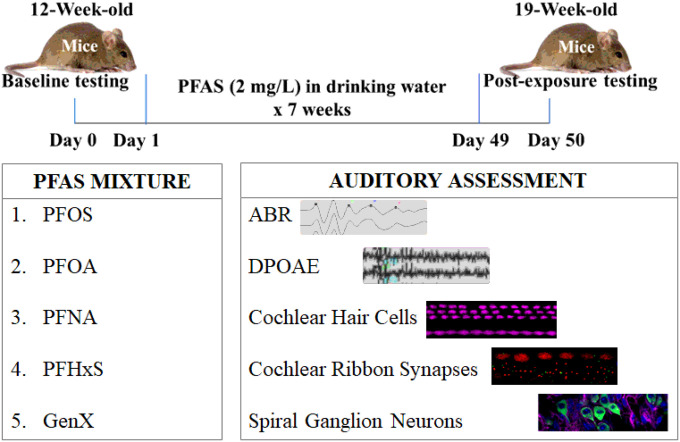
Overview of research design. Mice were exposed to a mixture of 5 PFAS each at an equal concentration of 2 mg/L in drinking water for 7 weeks. Hearing loss was assessed by measuring the shift in ABR thresholds, and outer hair cell activity was assessed by measuring DPOAE amplitudes. Hair cell loss, ribbon synapse disruption, and Spiral ganglion cell loss were assessed by immunohistochemical analysis of cochlear sensory epithelium.

In addition to the well-known toxic effects of PFAS on multiple organ systems, recent studies have indicated a possible association between exposure to PFAS and hearing loss [41, 42]. Analysis of data collected from 5,560 American adults indicated that 8.3% of the participants who had moderate hearing trouble also had higher urinary levels of hydrocarbons and polyfluorinated compounds [41]. Another study indicated that adults with very high levels (top 10%) of PFNA or PFDA had about 70–75% higher odds of high-frequency hearing impairment [43]. Higher serum PFOS is linked to a 9.75% increase in TSH, suggesting thyroid disruption [44]. Prenatal exposure to PFAS, especially PFOA and PFOS, has been associated with altered neonatal thyroid hormone levels [45]. In vitro, animal, and human studies show that various PFAS exposure can disrupt thyroid function, with increased risks of hypothyroidism observed in pregnant women and children [46]. Furthermore, congenital hypothyroidism, which is characterized by thyroid hormone deficiency, is a significant risk factor for both central and peripheral hearing disorders [47] and is strongly linked to auditory impairments [48]. Evidence shows that disrupted thyroid hormone signaling, such as reduced sensitivity of thyroid hormone receptor α (TRα1), can lead to early-onset high-frequency sensorineural hearing loss, outer hair cell (OHC) loss, and accelerated age-related hearing decline [49]. Since PFAS are known endocrine disruptors that interfere with thyroid hormone homeostasis, exposure during critical developmental windows may contribute to OHC damage and hearing loss. Moreover, several studies indicate that PFAS can activate peroxisome proliferator-activated receptors (PPARs) and disrupt essential cellular processes like growth and metabolism [50–54], which can potentially affect auditory function because PPARs are expressed in the OHCs and inner hair cells (IHCs), the sensory receptor cells in the cochlea. Despite these supportive evidences, the PFAS-induced auditory dysfunction is yet to be fully characterized. The present study aims to elucidate the effect of PFAS exposure on cochlear structure and function in a mouse model (Fig. 1).

## Results

### PFAS exposure induces a shift in hearing thresholds

**Figure 2:**
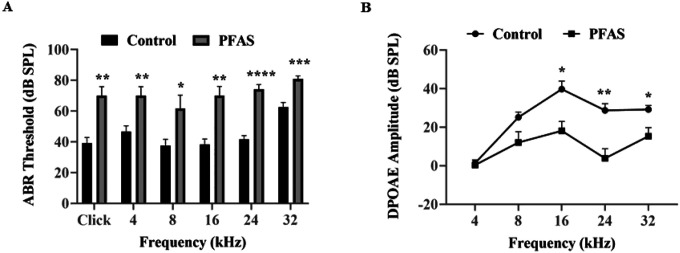
PFAS-induced changes in hearing thresholds and outer hair cell activity. **(A)** ABRs demonstrate that PFAS exposure elevated the hearing thresholds by 18 to 33 dB, with data collected from the left ear presented.. The results are expressed as Mean ± SEM; n = 6. * indicates p < 0.05, **p < 0.01, ***p < 0.001 and ****p < 0.0001 vs control. **(B)** The DPOAEs recorded using 4, 8, 16, 24, as well as 32 kHz f2 stimuli elicited at 70 dB SPL (L1) suggest that PFAS exposure decreased the DPOAE amplitudes at 16 kHz, 24 kHz, and 32 kHz frequency regions. Data are presented as Mean ± SEM; n = 4. * indicates p < 0.05, **p < 0.01vs control. ABR, auditory brainstem response; DPOAE, distortion product otoacoustic emissions.

The effect of PFAS on hearing thresholds was evaluated through ABR measurements. Mice exposed to PFAS-contaminated drinking water for a period of seven weeks showed notable elevations in hearing thresholds, with increases between 18 and 33 dB observed at various frequencies (4–32 kHz) as well as in response to click stimuli, as compared to control mice (Fig. 2A). These results demonstrate that exposure to PFAS leads to substantial hearing deficit in mice.

### PFAS exposure induces outer hair cell loss

**Figure 3:**
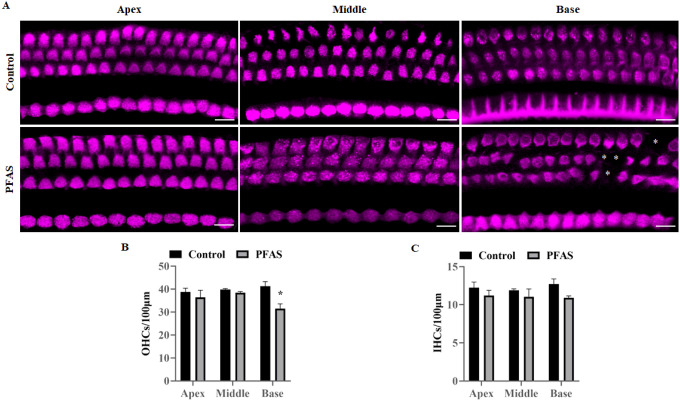
Effect of PFAS on cochlear hair cells. **(A)** Representative images of immunolabeled apex, middle, and basal regions of the cochlea using anti-myosin VIIa illustrate a loss of OHCs in the basal turn of the cochlea after PFAS exposure (indicated by an asterisk). Images are representative of three independent biological replicates. Scale bar = 20 μm. **(B, C)** The OHCs and IHCs counted per 100 μm section of the apex, middle, and basal region of the cochlea are shown. Quantification indicates a significant reduction in OHC counts after PFAS exposure. No IHC loss occurred after PFAS exposure. Data are presented asMean ± SEM; n = 3. * indicates p < 0.05 vs control. OHCs, outer hair cells; IHC, inner hair cells.

DPOAEs, a sensitive indicator of OHC function, showed a significant decrease in DPOAE amplitudes at 16 (p < 0.05), 24 (p < 0.01), and 32 kHz (p < 0.05) in PFAS-exposed animals (Fig. 2B). These deficits were frequency-specific, with more pronounced effects in the high-frequency cochlear regions. Immunolabeling of cochlear surface preparations using anti-myosin VIIa revealed a region-specific loss of OHCs localized to the basal turn of the cochlea (Fig. 3A). Furthermore, quantification of hair cells confirmed a ~ 24% loss of OHC (p < 0.05) in this region, while IHCs remained largely unaffected (Fig. 3B–C).

### PFAS exposure disrupts the ribbon synapses and induces cochlear synaptopathy

**Figure 4:**
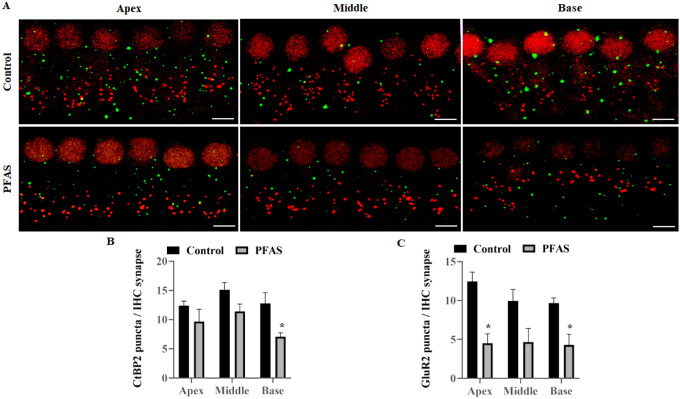
PFAS-induced changes in the cochlear ribbon synapses. **(A)** Immunohistochemical staining of ribbon synapses in the apex, middle, and basal turn of the cochlea indicated that PFAS exposure affected both the presynaptic ribbons and postsynaptic receptors. The presynaptic ribbons were stained with anti-CtBP2 (red), and postsynaptic receptors were stained with anti-GluR2 (green). The images are representative of three independent biological replicates, scale bar = 10 μm. **(B, C)**, Quantification of the CtBP2 and GluR2 puncta per IHC synapse indicated that PFAS exposure significantly decreased the number of CtBP2 puncta in the basal turn, while GluR2 puncta in the apical and basal turn of the organ of Corti. Data are presented as Mean ± SEM; n = 3. * indicates p < 0.05 vs control. CtBP2, C-terminal-binding protein-2; GluR2, glutamate receptor 2; IHC, inner hair cell.

**Figure 5:**
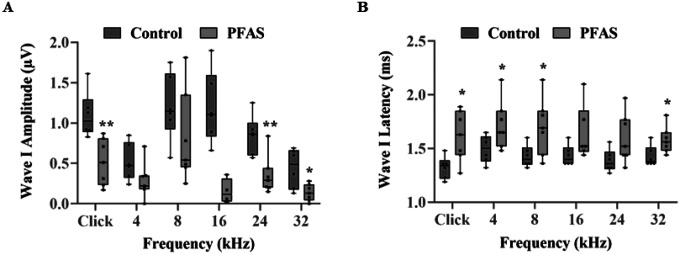
Effect of PFAS on the signal transmission in cochlear nerve fibers. **(A)** PFAS exposure significantly reduced the amplitude of ABR wave I elicited using click, as well as 24, and 32 kHz stimulus frequencies, suggesting a decrease in the number of neurons firing in response to a stimulus. The PFAS-induced changes were greater at the higher frequencies, indicating that the basal region is more susceptible to PFAS-induced toxicity. Data are presented as Mean ± SEM; n = 6–7. * indicates p < 0.05 and **p < 0.01 vs control. **(B)** PFAS exposure increased the latency of ABR wave I elicited using click, as well as 4, 8, and 32 kHz frequencies, which suggests that the speed of neural conduction is affected. Data are presented as Mean ± SEM; n = 6–7. * indicates p < 0.05 vs control. ABR, auditory brainstem response.

The disruption of cochlear ribbon synapses following PFAS exposure was investigated using immunohistochemical staining of the presynaptic ribbon marker CtBP2 and the postsynaptic receptor marker GluR2 (Fig. 4A). Quantitative analysis revealed ~ 39% decrease in CtBP2 puncta in the basal cochlear region, indicating loss of presynaptic ribbon synapses, while GluR2 puncta showed a more extensive reduction, ~ 64% in the apical and ~ 56% in the basal regions, suggesting widespread postsynaptic damage (Figs. 4B–C). These findings suggest that PFAS-induced disruption of ribbon synapses may contribute to auditory deficits. Functional consequences of these synaptic changes were assessed by ABR wave I, reflecting auditory nerve fiber synchrony. PFAS-exposed animals exhibited a significant reduction in ABR wave I amplitudes at 24 kHz (p < 0.01), 32 kHz (p < 0.05), and click stimuli (p < 0.01), indicating decreased neuronal firing (Fig. 5A). Additionally, wave I latencies were significantly prolonged at 4, 8, and 32 kHz frequencies as well as click stimuli (p < 0.05), suggesting delayed signal transmission (Fig. 5B). Together, these structural and functional alterations are indicative of PFAS-induced cochlear synaptopathy.

### PFAS exposure induces loss of spiral ganglions

**Figure 6:**
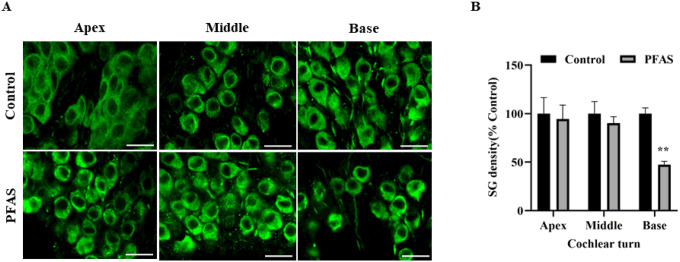
Effect of PFAS on spiral ganglion neurons. **(A)** Immunohistochemical analysis with β3-tubulin (green) indicated loss of SGNs after PFAS exposure in the basal turn of the cochlea. Images are representative of three independent biological replicates. Scale bar = 20 μm. **(B)** A count of the SGNs revealed a significant reduction in SG density in the basal turn of the cochlea after PFAS exposure. Data are presented asmean ± SEM; n = 3. ** indicates p < 0.01 vs control. SGNs, spiral ganglion neurons.

The neurodegenerative effects of PFAS exposure on the cochlea were assessed by immunolabeling spiral ganglion neurons (SGNs) using β3-tubulin. Mice exposed to PFAS exhibited a notable loss of SGNs, especially in the basal region of the cochlea, compared to control animals (Fig. 6A). Quantitative assessment showed ~ 45% decrease in SGN density (p < 0.01), indicating that PFAS-induced neurotoxicity likely plays a significant role in the observed hearing deficits (Fig. 6B).

## Discussion

This study provides the first experimental evidence that chronic exposure to a mixture of environmentally relevant PFAS induces both structural and functional damage in the auditory system. Mice exposed to PFAS-contaminated drinking water exhibited significantly elevated ABR thresholds across a wide frequency range (4–32 kHz), indicating sensorineural hearing loss. Functional deficits in OHCs, evidenced by reduced DPOAEs, were accompanied by region-specific OHC loss in the basal turn of the cochlea. In addition, immunohistochemical analyses demonstrated disruption of cochlear ribbon synapses and a marked decline in spiral ganglion neuron (SGN) density. These morphological alterations were accompanied by significant reductions in ABR wave I amplitudes and prolonged latencies, reflecting impaired auditory nerve synchrony and PFAS-induced cochlear synaptopathy. Our findings identify OHCs, ribbon synapses, and SGNs as key cochlear targets in PFAS-induced auditory dysfunction.

Epidemiological studies, including data from the National Health and Nutrition Examination Survey (NHANES), have reported positive correlations between serum levels of PFAS compounds such as PFOA, PFOS, PFNA, and PFHxS and hearing impairment [42]. However, most previous studies have examined the effects of individual PFAS compounds [42, 43], with limited investigation into PFAS mixtures, which more accurately reflect real-world exposure. The present study evaluates the ototoxic potential of a PFAS mixture in a mouse model, revealing that combined exposure can significantly impact cochlear structure and function. As prior studies have investigated the systemic effects of PFAS mixtures, including PFOA, PFOS, PFHxS, PFNA, and GenX, in Ldlr^−/−^ mice [55], we used the same strain as well as the same exposure paradigm to assess auditory outcomes in this study. After seven weeks of PFAS exposure, the average circulating concentrations in female mice was found to be 21.6 μg/mL (PFOA), 20.1 μg/mL (PFOS), 31.2 μg/mL (PFHxS), 23.5 μg/mL (PFNA), and 1.5 μg/mL (GenX), while slightly lower levels observed in males [55]. Data generated from both male and female mice were analyzed together because sex-related variability in the primary outcomes was not significant. The observed PFAS concentrations, though higher than those typically observed in the general population, fall within the range reported in highly exposed human cohorts, including industrial workers, individuals residing near PFAS-contaminated sites (e.g., areas affected by aqueous film-forming foam [AFFF] spills), and communities with contaminated drinking water [55–57]. The exposure paradigm used in this study has resulted in significant PFAS accumulation in mouse circulation, indicative of a substantial body burden and providing a relevant model for high-exposure human scenarios. Importantly, structural and functional auditory deficits observed in PFAS-exposed mice strongly align with previous epidemiological reports, reinforcing the hypothesis that PFAS compounds impair auditory function [42, 43].

OHCs are crucial for amplifying sound signals, and dysfunction of these cells can result in significant hearing deficits. Notably, the hearing threshold shifts and the OHC loss indicate that the damage was more pronounced in the basal turn of the cochlea, suggesting that this region is more susceptible to PFAS-induced ototoxicity. As PFAS exposure disrupts thyroid hormone homeostasis [58–62], which plays an essential role in the development and maintenance of the auditory system [48, 63], the observed auditory deficits may be due to PFAS-induced disruption of thyroid hormone homeostasis. PFAS also activates PPARs, which can modulate cell proliferation, differentiation, and lipid and glucose homeostasis [51–54]. As PPARs are present in both OHCs and IHCs [50], PFAS exposure can potentially affect the function and survival of these hair cells through the activation of PPAR. Furthermore, exposure to PFAS is associated with increased oxidative and nitrative stress [64, 65]. As cochlear oxidative and nitrative stress leads to apoptosis of auditory hair cells [66] and damages other structures within the cochlea, resulting in hearing loss [67, 68], the observed significant loss of OHCs following PFAS exposure could be attributed to potential PFAS-induced increase in the oxidative/nitrative stress in the cochlea.

The ribbon synapses are crucial in transmitting signals from the IHCs to the auditory neurons and serve as the primary excitatory afferent synapses within the auditory pathway [69, 70]. Ribbon synapses enable continuous and precise release of neurotransmitters at these synapses, which governs the firing pattern of the auditory nerve and determines sound intensity and timing [71, 72]. Their disruption can impair sensory processing and lead to the development of cochlear synaptopathy [73]. The ABR wave I amplitudes and latencies are considered an indicator of ribbon-synapse functionality [74]. A noticeable decrease in the amplitude of ABR wave-I and an increase in wave-I latency was observed in PFAS-exposed animals compared to the control group, suggesting a reduction in the number of neurons firing and a delay in the response to sound stimuli, leading to cochlear synaptopathy. Exposure to PFAS such as PFOS and PFOA disrupts neurotransmission by inhibiting GABA_A_ receptor function [75], altering glutamate-activated and potassium currents [76, 77], and impairing synaptic calcium homeostasis by elevating intracellular calcium in exposed neurons [37]. Additionally, PFAS-induced oxidative stress can damage synaptic structures and impair neurotransmission [78, 79]. Consistent with these reports, immunohistochemistry analysis revealed that PFAS exposure significantly damaged outer OHCs, ribbon synapses, and SGNs, particularly in high-frequency regions of the cochlea. Notably, PFAS exposure led to a marked reduction in SGNs density, providing evidence of PFAS-induced neuronal damage in the cochlea. These findings are in agreement with other reports that indicate that PFAS disrupts neuronal integrity through mechanisms involving oxidative stress, dysregulated lipid metabolism, and impaired synaptic signaling [33, 80, 81]. The susceptibility of cochlear ribbon synapses to environmental insults was also noticed in recent studies on the complex molecular changes underlying lead-induced cochlear synaptopathy [82]. Thus, the PFAS-induced damage to the cochlea potentially contributes to the auditory deficits observed following PFAS exposure.

Collectively, these findings provide the first experimental evidence identifying critical cochlear targets in PFAS-induced auditory dysfunction. Exposure to PFAS mixture induced significant damage to OHCs, ribbon synapses, and SGNs in the cochlea, underscoring the importance of real-world exposures, which often involve combinations of multiple PFAS compounds. Further studies are needed to elucidate the molecular mechanisms underlying PFAS-induced cochlear damage and discover effective interventions to mitigate PFAS-induced auditory dysfunction.

## Materials and methods

### Experimental design and statistical rationale

Auditory impairments were examined in mice subjected to PFAS exposure (Fig. 1). The study design included male and female mice in every experimental set, and the data from these sexes were combined for analysis. Electrophysiological analyses used four to seven biological replicates, while immunohistochemistry analyses included at least three biological replicates.

### Animals

Male and female B6.129S7-Ldlrtm1Her/J mice (strain # 002207, weighing between 18 and 24 grams) on a C57BL/6J genetic background were obtained from Jackson Laboratories at seven weeks of age and were allowed to acclimate for one week upon arrival. The mice were exposed to a mixture of five PFAS in their drinking water for seven weeks. The PFAS mixture contained equal concentrations (2 mg/L each) of the following compounds: perfluorooctane sulfonate (PFOS; Sigma-Aldrich # 2795-39-3, ≥ 98.0%), perfluorooctanoic acid (PFOA; Sigma-Aldrich # 335-67-1, 95%), perfluorononanoic acid (PFNA; Sigma-Aldrich # 375-95-1, 97%), tridecafluorohexane-1-sulfonic acid (PFHxS; J&K Scientific # 3871-99-6, ≥ 98.0%), and ammonium perfluoro(2-methyl-3-oxahexanoate) (GenX; Synquest Laboratories, CAS No. 62037-80-3, 95%).. Control group mice were provided with normal drinking water, and the water bottles were replaced on a weekly basis. All animal procedures were approved by the Institutional Animal Care and Use Committee (IACUC) at Wayne State University. All experiments were performed following the relevant guidelines and regulations and are reported as per the ARRIVE guidelines. Mice were euthanized using carbon dioxide inhalation.

### Auditory brainstem responses and distortion product otoacoustic emissions

Auditory brainstem responses (ABRs) and distortion product otoacoustic emissions (DPOAEs) were recorded at baseline and post-exposure to lead and noise. Sound stimulus presentation and data acquisition were conducted using the Tucker-Davis Technologies (TDT) System III hardware platform, with BioSigRZ software (version 5.7.6). Mice were anesthetized with isoflurane (4% for induction, followed by 1.5% for maintenance) using an oxygen flow rate of 1 L/min and positioned on a temperature-controlled heating pad (38°C) inside a sound-attenuated chamber (Model ECKEL-AB-4230, Morrisburg, Ontario, Canada). Subdermal needle electrodes were placed at the left mastoid region (reference), the vertex of the skull (active), and the right ear (ground). Acoustic stimuli, consisting of clicks and tone bursts at frequencies of 4, 8, 16, 24, and 32 kHz, were delivered to the external auditory canal. For each stimulus type, waveforms were obtained by averaging responses from 512 presentations delivered at a rate of 21 stimuli per second. Stimulus intensity was decreased in 5 dB increments starting from 90 dB SPL until no ABR response was detectable. The ABR threshold was determined as the lowest intensity level that evoked a Wave I response. Wave I amplitude, measured as the voltage difference between the peak and trough, and latency, time from stimulus onset to the peak, were analyzed at 90 dB SPL.

The DPOAE test used two primary tones, f1 and f2, with a fixed ratio of f2/f1 = 1.2. The level difference (L2–L1) was + 10 dB, and L1 intensity decreased from 80 dB SPL to 20 dB SPL in 10 dB steps. Stimuli were generated by the TDT RZ6 system and delivered through TDT multifield magnetic speakers (Alachua, FL, USA). The f2 frequency ranged from 4 kHz to 32 kHz. Distortion product emissions at 2f1–f2 were recorded using an ER10B + probe microphone (Etymotic Research, Elk Grove Village, IL, USA) with TDT equipment. Data were collected every 20.971 ms and averaged over 512 repetitions.

### Immunohistochemistry of cochlear surface preparations

Following euthanasia, the temporal bones were extracted and immersed in ice-cold phosphate-buffered saline (PBS, pH 7.4) immediately. The cochleae were perfused through the oval window with 4% paraformaldehyde (PFA) in PBS to flush out blood and initiate fixation, then immersed in the same fixative overnight at 4°C. Decalcification step was performed in 100 mM ethylenediaminetetraacetic acid (EDTA) in PBS at 4°C for 48–72 hours to soften the bony capsule. Using a stereomicroscope (Leica model # M 165 C), the cochlear sensory epithelium was carefully microdissected from surrounding structures and separated into apical, middle, and basal turns. For immunolabeling, tissues were blocked with 10% normal goat serum (Thermofisher Scientific # 50062Z) in PBS containing Triton X-100 and bovine serum albumin for one hours at room temperature to reduce non-specific binding, followed by overnight incubation at 4°C with primary antibodies such as mouse anti-myosin VIIa antibody (IgG2a, 1:400, Santa Cruz biotechnology # 74516), mouse anti-C-terminal-binding protein-2 (CtBP2; IgG1, 1:200, BD Biosciences # 612044), rabbit anti-glutamate receptor 2 (GluR2; IgG, 1:200, Thermo Fisher Scientific # PA5–79326), mouse anti-nitrotyrosine antibody (IgG2a κ, 1:200, Santa Cruz biotechnology # sc-32757), rabbit anti-tubulin β−3 (TUBB3) antibody (IgG, 1:100, BioLegend # 802001). After thorough washes, appropriate Alexa Fluor-conjugated secondary antibodies used were goat anti-rabbit Alexa Fluor (AF) 647 (IgG, 1:400, Thermo Fisher Scientific # A-21244), goat anti-mouse AF 568 (IgG2a, 1:400, Thermo Fisher Scientific # A-21134), and goat anti-mouse AF 488 (IgG2a, 1:400, Thermo Fisher Scientific # A-21131) and AF 488-conjugated phalloidin (1:150, Thermo Fisher Scientific # A-12379). Tissue mounting was done in ProLong Gold Antifade Mountant containing DAPI (Thermo Fisher Scientific # P36935), and coverslips were secured along the edges using a coverslip sealant (Thermo Fisher Scientific # NC0154994).

### Confocal microscopy and image processing

Imaging was performed using a Zeiss LSM 800 confocal microscope with a 40x objective (NA 1.3) to capture outer hair cells (OHCs), inner hair cells (IHCs), and spiral ganglion neurons (SGNs), and a 63x objective (NA 1.4) for presynaptic (CtBP2) and postsynaptic (GluR2) structures. Z-stacks of 10–15 slices for hair cells and 20–25 slices for synaptic puncta were acquired and processed into maximum intensity projections. Immune-labeled hair cells were quantified per 100 μm cochlear length, while SGNs labeled with β-III tubulin were counted over 10,000 μm^2^ areas. Nitrotyrosine (3-NT) intensity was measured in both SGNs and OHCs. All analyses were done using Zeiss Zen Blue 3.7.

### Statistical analysis

Statistical analyses were conducted with using GraphPad Prism (v10.2.3, La Jolla, CA, USA) and Microsoft Excel (Microsoft 365, v2404, Redmond, WA, USA) and. To evaluate differences among groups, a two-tailed unpaired t-test was employed, with statistical significance set at p < 0.05.. Results are expressed as mean ± standard error of the mean.

## Data Availability

The datasets used and analyzed in the current study will be made available on request.

## References

[1] Oh J. Childhood exposure to per- and polyfluoroalkyl substances and neurodevelopment in the CHARGE case-control study. Environ. Res. 215, 114322 (2022). 36108719 10.1016/j.envres.2022.114322 PMC9976729

[2] U.S. Environmental Protection Agency. Our current understanding of the human health and environmental risks of PFAS. (2024). https://www.epa.gov/pfas/our-current-understanding-human-health-and-environmental-risks-pfas

[3] ATSDR (Agency for Toxic Substances and Disease Registry). Health effects of PFAS. (2024). https://www.atsdr.cdc.gov/pfas/about/health-effects.html

[4] NIEHS (National Institute of Environmental Health Sciences.). Per- and polyfluoroalkyl substances (PFAS). (2024). https://www.niehs.nih.gov/health/topics/agents/pfc

[5] Clara M., Scheffknecht C., Scharf S., Weiss S. & Gans O. Emissions of perfluorinated alkylated substances (PFAS) from point sources—identification of relevant branches. Water Sci. Technol. 58, 59–66 (2008). 18653937 10.2166/wst.2008.641

[6] Nickerson A. Enhanced extraction of AFFF-associated PFASs from source zone soils. Environ. Sci. Technol. 54, 4952–4962 (2020). 32200626 10.1021/acs.est.0c00792

[7] Trier X., Granby K. & Christensen J. H. Polyfluorinated surfactants (PFS) in paper and board coatings for food packaging. Environ. Sci. Pollut Res. Int. 18, 1108–1120 (2011). 21327544 10.1007/s11356-010-0439-3

[8] Drage D. S., Sharkey M., Berresheim H., Coggins M. & Harrad S. Rapid determination of selected PFAS in textiles entering the waste stream. Toxics 11, 55 (2023). 36668781 10.3390/toxics11010055 PMC9860823

[9] Buck R. C. Perfluoroalkyl and polyfluoroalkyl substances in the environment: terminology, classification, and origins. Integr. Environ. Assess. Manag. 7, 513–541 (2011). 21793199 10.1002/ieam.258 PMC3214619

[10] Sadia M. Forever legacies? Profiling historical PFAS contamination and current influence on groundwater used for drinking water. Sci. Total Environ. 890, 164420 (2023). 37236451 10.1016/j.scitotenv.2023.164420

[11] Domingo J. L. & Nadal M. Per- and polyfluoroalkyl substances (PFASs) in food and human dietary intake: a review of the recent scientific literature. J. Agric. Food Chem. 65, 533–543 (2017). 28052194 10.1021/acs.jafc.6b04683

[12] Liddie J. M., Schaider L. A. & Sunderland E. M. Sociodemographic factors are associated with the abundance of PFAS sources and detection in U.S. community water systems. Environ. Sci. Technol. 57, 7902–7912 (2023). 37184106 10.1021/acs.est.2c07255 PMC10233791

[13] Calafat A. M., Wong L. Y., Kuklenyik Z., Reidy J. A. & Needham L. L. Polyfluoroalkyl chemicals in the U.S. population: data from the National Health and Nutrition Examination Survey (NHANES) 2003–2004 and comparisons with NHANES 1999–2000. Environ. Health Perspect. 115, 1596–1602 (2007). 18007991 10.1289/ehp.10598 PMC2072821

[14] Bline A. P. Public health risks of PFAS-related immunotoxicity are real. Curr. Environ. Health Rep. 11, 118–127 (2024). 38526771 10.1007/s40572-024-00441-y PMC11081924

[15] Jensen A. A. & Leffers H. Emerging endocrine disrupters: perfluoroalkylated substances. Int. J. Androl. 31, 161–169 (2008). 18315716 10.1111/j.1365-2605.2008.00870.x

[16] Qazi M. R., Abedi M. R., Nelson B. D., DePierre J. W. & Abedi-Valugerdi M. Dietary exposure to perfluorooctanoate or perfluorooctane sulfonate induces hypertrophy in centrilobular hepatocytes and alters the hepatic immune status in mice. Int. Immunopharmacol. 10, 1420–1427 (2010). 20816993 10.1016/j.intimp.2010.08.009

[17] Mariussen E. Neurotoxic effects of perfluoroalkylated compounds: mechanisms of action and environmental relevance. Arch. Toxicol. 86, 1349–1367 (2012). 22456834 10.1007/s00204-012-0822-6

[18] Barry V., Winquist A. & Steenland K. Perfluorooctanoic acid (PFOA) exposures and incident cancers among adults living near a chemical plant. Environ. Health Perspect. 121, 1313–1318 (2013). 24007715 10.1289/ehp.1306615 PMC3855514

[19] Vieira V. M. Perfluorooctanoic acid exposure and cancer outcomes in a contaminated community: a geographic analysis. Environ. Health Perspect. 121, 318–323 (2013). 23308854 10.1289/ehp.1205829 PMC3621179

[20] Gleason J. A., Post G. B. & Fagliano J. A. Associations of perfluorinated chemical serum concentrations and biomarkers of liver function and uric acid in the US population (NHANES), 2007–2010. Environ. Res. 136, 8–14 (2015). 25460614 10.1016/j.envres.2014.10.004

[21] Conway B. N., Badders A. N., Costacou T., Arthur J. M. & Innes K. E. Perfluoroalkyl substances and kidney function in chronic kidney disease, anemia, and diabetes. Diabetes Metab. Syndr. Obes. 11, 707–716 (2018). 30532572 10.2147/DMSO.S173809 PMC6244585

[22] Song X. Biomonitoring PFAAs in blood and semen samples: investigation of a potential link between PFAAs exposure and semen mobility in China. Environ. Int. 113, 50–54 (2018). 29421407 10.1016/j.envint.2018.01.010

[23] Jin R. Perfluoroalkyl substances and severity of nonalcoholic fatty liver in children: an untargeted metabolomics approach. Environ. Int. 134, 105220 (2020). 31744629 10.1016/j.envint.2019.105220 PMC6944061

[24] Lin P. D. Per- and polyfluoroalkyl substances and kidney function: follow-up results from the Diabetes Prevention Program trial. Environ. Int. 148, 106375 (2021). 33482440 10.1016/j.envint.2020.106375 PMC7929640

[25] Fenton S. E. Per- and polyfluoroalkyl substance toxicity and human health review: current state of knowledge and strategies for informing future research. Environ. Toxicol. Chem. 40, 606–630 (2021). 33017053 10.1002/etc.4890 PMC7906952

[26] Steenland K. & Winquist A. PFAS and cancer, a scoping review of the epidemiologic evidence. Environ. Res. 194, 110690 (2021). 33385391 10.1016/j.envres.2020.110690 PMC7946751

[27] ATSDR (Agency for Toxic Substances and Disease Registry). Toxicological profile for perfluoroalkyls. U.S. Department of Health and Human Services, Atlanta, GA. (2021). https://www.atsdr.cdc.gov/ToxProfiles/tp200.pdf 37220203

[28] Costello E. Exposure to per- and polyfluoroalkyl substances and markers of liver injury: a systematic review and meta-analysis. Environ. Health Perspect. 130, 046001 (2022). 35475652 10.1289/EHP10092 PMC9044977

[29] Batzella E. Perfluoroalkyl substance mixtures and cardio-metabolic outcomes in highly exposed male workers in the Veneto Region: a mixture-based approach. Environ. Res. 212, 113225 (2022). 35390304 10.1016/j.envres.2022.113225

[30] Xie Z. Associations between prenatal exposure to perfluoroalkyl substances and neurobehavioral development in early childhood: a prospective cohort study. Ecotoxicol. Environ. Saf. 241, 113818 (2022). 35777342 10.1016/j.ecoenv.2022.113818

[31] Wen Z. J., Wei Y. J. & Zhang Y. F. A review of cardiovascular effects and underlying mechanisms of legacy and emerging per- and polyfluoroalkyl substances (PFAS). Arch. Toxicol. 97, 1195–1245 (2023). 36947184 10.1007/s00204-023-03477-5

[32] Cao Y. & Ng C. Absorption, distribution, and toxicity of per- and polyfluoroalkyl substances (PFAS) in the brain: a review. Environ. Sci. : Process. Impacts. 23, 1623–1640 (2021). 34533150 10.1039/d1em00228g

[33] Brown-Leung J. M. & Cannon J. R. Neurotransmission targets of per- and polyfluoroalkyl substance neurotoxicity: mechanisms and potential implications for adverse neurological outcomes. Chem. Res. Toxicol. 35, 1312–1333 (2022). 35921496 10.1021/acs.chemrestox.2c00072 PMC10446502

[34] Dusza H. M. Effects of environmental pollutants on calcium release and uptake by rat cortical microsomes. Neurotoxicology 69, 266–277 (2018). 30056177 10.1016/j.neuro.2018.07.015

[35] Fang X., Wu C., Li H., Yuan W. & Wang X. Elevation of intracellular calcium and oxidative stress is involved in perfluorononanoic acid-induced neurotoxicity. Toxicol. Ind. Health. 34, 139–145 (2018). 29187073 10.1177/0748233717742262

[36] Liao C. Y., Li X. Y., Wu B., Duan S. & Jiang G. B. Acute enhancement of synaptic transmission and chronic inhibition of synaptogenesis induced by perfluorooctane sulfonate through mediation of voltage-dependent calcium channel. Environ. Sci. Technol. 42, 5335–5341 (2008). 18754390 10.1021/es800018k

[37] Liu X., Jin Y., Liu W., Wang F. & Hao S. Possible mechanism of perfluorooctane sulfonate and perfluorooctanoate on the release of calcium ion from calcium stores in primary cultures of rat hippocampal neurons. Toxicol. Vitro. 25, 1294–1301 (2011). 10.1016/j.tiv.2011.04.016 21575708

[38] Wang Y., Zhao H., Zhang Q., Liu W. & Quan X. Perfluorooctane sulfonate induces apoptosis of hippocampal neurons in rat offspring associated with calcium overload. Toxicol. Res. 4, 931–938 (2015).

[39] Qian Y. Perfluorooctane sulfonate (PFOS) induces reactive oxygen species (ROS) production in human microvascular endothelial cells: role in endothelial permeability. J. Toxicol. Environ. Health A. 73, 819–836 (2010). 20391123 10.1080/15287391003689317 PMC3107001

[40] Tan Y. Association between a mixture of per- and polyfluoroalkyl substances (PFAS) and inflammatory biomarkers in the Atlanta African American maternal-child cohort. Environ. Sci. Technol. 57, 13419–13428 (2023). 37649345 10.1021/acs.est.3c04688 PMC10900195

[41] Shiue I. Urinary heavy metals, phthalates, perchlorate, nitrate, thiocyanate, hydrocarbons, and polyfluorinated compounds are associated with adult hearing disturbance: USA NHANES, 2011–2012. Environ. Sci. Pollut Res. Int. 22, 20306–20311 (2015). 26490897 10.1007/s11356-015-5546-8

[42] Li M. C. Serum per- and polyfluoroalkyl substances are associated with increased hearing impairment: a re-analysis of the National Health and Nutrition Examination Survey data. Int. J. Environ. Res. Public. Health. 17, 5836 (2020). 32806617 10.3390/ijerph17165836 PMC7460726

[43] Ding N. & Park S. K. Perfluoroalkyl substances exposure and hearing impairment in US adults. Environ. Res. 187, 109686 (2020). 32474307 10.1016/j.envres.2020.109686 PMC7331829

[44] Blake B. E., Pinney S. M., Hines E. P., Fenton S. E. & Ferguson K. K. Associations between longitudinal serum perfluoroalkyl substance (PFAS) levels and measures of thyroid hormone, kidney function, and body mass index in the Fernald Community Cohort. Environ. Pollut. 242, 894–904 (2018). 30373035 10.1016/j.envpol.2018.07.042 PMC6309414

[45] Rosen Vollmar A. K. Per- and polyfluoroalkyl substances (PFAS) and thyroid hormone measurements in dried blood spots and neonatal characteristics: a pilot study. J. Expo Sci. Environ. Epidemiol. 33, 737–747 (2023). 37730931 10.1038/s41370-023-00603-4 PMC10541328

[46] Coperchini F. Thyroid disrupting effects of old and new generation PFAS. Front. Endocrinol. 11, 612320 (2021). 10.3389/fendo.2020.612320 PMC7851056 33542707

[47] Andrade C. L. O. A. D. Mechanisms involved in hearing disorders of thyroid ontogeny: a literature review. Arch. Endocrinol. Metab. 61, 501–505 (2017). 28977164 10.1590/2359-3997000000292 PMC10522256

[48] Andrade C. L. O., Alves C. A. D. & Ramos H. E. Congenital hypothyroidism and the deleterious effects on auditory function and language skills: a narrative review. Front. Endocrinol. 12, 671784 (2021). 10.3389/fendo.2021.671784 PMC8382885 34447350

[49] Affortit C. A disease-associated mutation in thyroid hormone receptor α1 causes hearing loss and sensory hair cell patterning defects in mice. Sci. Signal. 15, eabj4583 (2022). 10.1126/scisignal.abj4583 35700264

[50] Sekulic-Jablanovic M. Effects of peroxisome proliferator activated receptors (PPAR)-γ and -α agonists on cochlear protection from oxidative stress. PLoS One. 12, e0188596 (2017). 29182629 10.1371/journal.pone.0188596 PMC5705132

[51] Intrasuksri U., Rangwala S. M., O’Brien M., Noonan D. J. & Feller D. R. Mechanisms of peroxisome proliferation by perfluorooctanoic acid and endogenous fatty acids. Gen. Pharmacol. 31, 187–197 (1998). 9688458 10.1016/s0306-3623(98)00029-9

[52] Kennedy G. L. The toxicology of perfluorooctanoate. Crit. Rev. Toxicol. 34, 351–384 (2004). 15328768 10.1080/10408440490464705

[53] Rosen M. B. PPARα-independent transcriptional targets of perfluoroalkyl acids revealed by transcript profiling. Toxicol 387, 95–107 (2017). 10.1016/j.tox.2017.05.013 PMC6129013 28558994

[54] Shipley J. M. trans-activation of PPARalpha and induction of PPARalpha target genes by perfluorooctane-based chemicals. Toxicol. Sci. 80, 151–160 (2004). 15071170 10.1093/toxsci/kfh130

[55] Roth K. Exposure of Ldlr−/− mice to a PFAS mixture and outcomes related to circulating lipids, bile acid excretion, and the intestinal transporter ASBT. Environ. Health Perspect. 132, 87007 (2024). 39177951 10.1289/EHP14339 PMC11343043

[56] Gu J. K. Serum concentration of selected per- and polyfluoroalkyl substances (PFAS) by industry and occupational groups among US adult workers, NHANES 2005–2014. Am. J. Ind. Med. 68, 531–542 (2025). 40293335 10.1002/ajim.23726 PMC12333454

[57] Mitchell C. L. Differences in serum concentrations of per- and polyfluoroalkyl substances by occupation among firefighters, other first responders, healthcare workers, and other essential workers in Arizona, 2020–2023. J. Expo Sci. Environ. Epidemiol. 35, 437–444 (2025). 40050415 10.1038/s41370-025-00753-7 PMC12068973

[58] Ballesteros V. Exposure to perfluoroalkyl substances and thyroid function in pregnant women and children: a systematic review of epidemiologic studies. Environ. Int. 99, 15–28 (2017). 27884404 10.1016/j.envint.2016.10.015

[59] Coperchini F. Thyroid disruption by perfluorooctane sulfonate (PFOS) and perfluorooctanoate (PFOA). J. Endocrinol. Investig. 40, 105–121 (2017). 27837466 10.1007/s40618-016-0572-z

[60] Kim M. J. Association between perfluoroalkyl substances exposure and thyroid function in adults: a meta-analysis. PLoS One. 13, e0197244 (2018). 29746532 10.1371/journal.pone.0197244 PMC5945046

[61] La Rocca C. Exposure and effective dose biomarkers for perfluorooctane sulfonic acid (PFOS) and perfluorooctanoic acid (PFOA) in infertile subjects: preliminary results of the PREVIENI project. Int. J. Hyg. Environ. Health. 215, 206–211 (2012). 22197512 10.1016/j.ijheh.2011.10.016

[62] Long M., Ghisari M. & Bonefeld-Jørgensen E. C. Effects of perfluoroalkyl acids on the function of the thyroid hormone and the aryl hydrocarbon receptor. Environ. Sci. Pollut Res. Int. 20, 8045–8056 (2013). 23539207 10.1007/s11356-013-1628-7

[63] Sharlin D. S., Visser T. J. & Forrest D. Developmental and cell-specific expression of thyroid hormone transporters in the mouse cochlea. Endocrinology 152, 5053–5064 (2011). 21878515 10.1210/en.2011-1372 PMC3230046

[64] Kamendulis L. M., Wu Q., Sandusky G. E. & Hocevar B. A. Perfluorooctanoic acid exposure triggers oxidative stress in the mouse pancreas. Toxicol. Rep. 1, 513–521 (2014). 28962265 10.1016/j.toxrep.2014.07.015 PMC5598264

[65] Wang X. Serum metabolome biomarkers associate low-level environmental perfluorinated compound exposure with oxidative/nitrosative stress in humans. Environ. Pollut. 229, 168–176 (2017). 28599201 10.1016/j.envpol.2017.04.086

[66] Jamesdaniel S., Rosati R., Westrick J. & Ruden D. M. Chronic lead exposure induces cochlear oxidative stress and potentiates noise-induced hearing loss. Toxicol. Lett. 292, 175–180 (2018). 29746905 10.1016/j.toxlet.2018.05.004 PMC6131708

[67] Shahab M. & Jamesdaniel S. Nitrative stress and auditory dysfunction. Pharmaceuticals 15, 649 (2022). 35745568 10.3390/ph15060649 PMC9227425

[68] Maniaci A. Hearing loss and oxidative stress: a comprehensive review. Antioxidants 13, 842 (2024). 39061910 10.3390/antiox13070842 PMC11274311

[69] Becker L. The presynaptic ribbon maintains vesicle populations at the hair cell afferent fiber synapse. eLife 7, e30241 (2018). 29328021 10.7554/eLife.30241 PMC5794257

[70] Gao L. Insulin-like growth factor 1 on the maintenance of ribbon synapses in mouse cochlear explant cultures. Front. Cell. Neurosci. 14, 571155 (2020). 33132846 10.3389/fncel.2020.571155 PMC7579230

[71] Fuchs P. A. Time and intensity coding at the hair cell’s ribbon synapse. J. Physiol. 566, 7–12 (2005). 15845587 10.1113/jphysiol.2004.082214 PMC1464726

[72] Johnson S. L., Safieddine S., Mustapha M. & Marcotti W. Hair cell afferent synapses: function and dysfunction. Cold Spring Harb Perspect. Med. 9, a033175 (2019). 30617058 10.1101/cshperspect.a033175 PMC6886459

[73] Wei M. Protection of cochlear ribbon synapses and prevention of hidden hearing loss. Neural Plast. 1–11 (2020). (2020). 10.1155/2020/8815990 PMC7652619 33204247

[74] Yuan X. Ribbon synapses and hearing impairment in mice after in utero sevoflurane exposure. Drug Des. Devel Ther. 14, 2685–2693 (2020). 10.2147/DDDT.S253031 PMC7354911 32753847

[75] Tukker A. M. Perfluorooctane sulfonate (PFOS) and perfluorooctanoate (PFOA) acutely affect human α1β2γ2L GABAA receptor and spontaneous neuronal network function in vitro. Sci. Rep. 10, 5311 (2020). 32210279 10.1038/s41598-020-62152-2 PMC7093421

[76] Liao C. Y., Cui L., Zhou Q. F., Duan S. M. & Jiang G. B. Effects of perfluorooctane sulfonate on ion channels and glutamate-activated current in cultured rat hippocampal neurons. Environ. Toxicol. Pharmacol. 27, 338–344 (2009). 21783962 10.1016/j.etap.2008.11.013

[77] Liao C. Changes in synaptic transmission, calcium current, and neurite growth by perfluorinated compounds are dependent on the chain length and functional group. Environ. Sci. Technol. 43, 2099–2104 (2009). 19368220 10.1021/es802985e

[78] Chen N., Li J., Li D., Yang Y. & He D. Chronic exposure to perfluorooctane sulfonate induces behavior defects and neurotoxicity through oxidative damages, in vivo and in vitro. PLoS ONE. 9, e113453 (2014). 25412474 10.1371/journal.pone.0113453 PMC4239059

[79] Sun P. Nrf2 signaling elicits a neuroprotective role against PFOS-mediated oxidative damage and apoptosis. Neurochem Res. 43, 2446–2459 (2018). 30382449 10.1007/s11064-018-2672-y

[80] Running L. Investigating the mechanism of neurotoxic effects of PFAS in differentiated neuronal cells through transcriptomics and lipidomics analysis. ACS Chem. Neurosci. 15, 4568–4579 (2024). 39603830 10.1021/acschemneuro.4c00652

[81] Li S. Adverse outcome pathway for the neurotoxicity of per- and polyfluoroalkyl substances: a systematic review. Eco-Environment Health. 3, 476–493 (2024). 39605965 10.1016/j.eehl.2024.08.002 PMC11599988

[82] Bhatia P. Unraveling the molecular landscape of lead-induced cochlear synaptopathy: a quantitative proteomics analysis. Front. Cell. Neurosci. 18, 1408208 (2024). 39104440 10.3389/fncel.2024.1408208 PMC11298392

